# P-1084. Antimicrobial Activity of Aztreonam-Avibactam and Other β-Lactamase Inhibitor Combinations Tested against Enterobacterales Isolates from Pediatric Patients from United States Medical Centers (2019–2023)

**DOI:** 10.1093/ofid/ofae631.1272

**Published:** 2025-01-29

**Authors:** Helio S Sader, Marisa Winkler, Rodrigo E Mendes, Mariana Castanheira

**Affiliations:** JMI Laboratories, North Liberty, Iowa; Element Materials Technology/Jones Microbiology Institute, North Liberty, Iowa; JMI Laboratories, North Liberty, Iowa; JMI Laboratories, North Liberty, Iowa

## Abstract

**Background:**

We evaluated the activities of aztreonam-avibactam (ATM-AVI), ceftazidime-avibactam (CAZ-AVI), meropenem-vaborbactam (MEM-VAB), imipenem-relebactam (IMI-REL), ceftolozane-tazobactam (C-T), and comparators against Enterobacterales (ENT) isolates causing infection in pediatric (PED) patients.

Activity of β-lactamase inhibitor combinations against Enterobacterales isolates from pediatric patients

**Figure:**
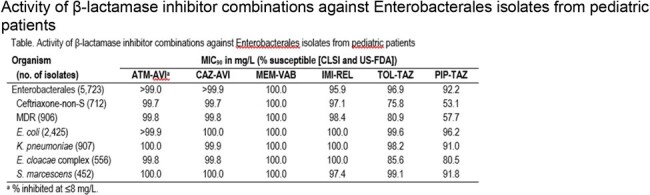

**Methods:**

Among 43,325 ENT (1/patient) collected in 2019–2023 via the INFORM Surveillance Program, 5,723 (13.2%) were from PED patients (≤ 17 years old [yo]). The isolates were consecutively collected from 81 US medical centers and susceptibility tested by CLSI broth microdilution method. Susceptibility was stratified by infection type and patient age: ≤1 yo (*n*=2,275), 2–5 yo (*n*=1,130), 6–12 yo (*n*=1,213), and 13–17 yo (*n*=1,105) and compared to adults (18–64 yo; *n*=17,712).

**Results:**

PED isolates were mainly from pneumonia (21.8%), bloodstream (BSI; 15.3%), and urinary tract infection (UTI; 51.8%). ATM-AVI inhibited > 99.9% of PED isolates at ≤ 8 mg/L; CAZ-AVI and MEM-VAB were active against > 99.9% and 100.0%, respectively, whereas IMI-REL and ceftolozane-tazobactam (TOL-TAZ) were slightly less active (Table). Meropenem, ceftriaxone (CRO), and gentamicin were active against 99.8%, 87.6%, and 92.1% of PED isolates, respectively. CRO susceptibility ranged from 84.5% (6–12 yo) to 89.2% (2–5 and 13–17 yo) among PED isolates and was 81.5% against adult isolates. CRO was active against 86.0%, 87.3%, and 88.4% of PED isolates from pneumonia, BSI, and UTI, respectively. Multidrug-resistant (MDR; non-susceptible [NS] to ≥ 3 classes) phenotype varied from 14.3% (13–17 yo) to 19.7% (6–12 yo) among PED isolates (15.8% overall) and was 20.7% among adult ENT. Carbapenem resistance rate was markedly lower among PED (0.1%) compared to adult isolates (1.3%). TOL-TAZ and piperacillin-tazobactam (PIP-TAZ) exhibited limited activity against CRO-NS and MDR isolates. Susceptibility rates for most comparators were higher among PED than adult isolates, including CRO (87.6% vs. 81.5%), PIP-TAZ (92.2% vs. 88.0%), TOL-TAZ (96.9% vs. 93.9%), and levofloxacin (90.8% vs. 82.1%).

**Conclusion:**

ATM-AVI, CAZ-AVI, and MEM-VAB were highly active against ENT isolates from PED patients. Susceptibility differences were observed among age groups and infection types.

**Disclosures:**

**Marisa Winkler, MD, PhD**, Element Iowa City (JMI Laboratories) was contracted to perform services in 2023 for > 30 biotech and pharmaceutical companies: Grant/Research Support **Rodrigo E. Mendes, PhD**, GSK: Grant/Research Support

